# Genetic diversity in populations of asexual and sexual bag worm moths (Lepidoptera: Psychidae)

**DOI:** 10.1186/1472-6785-5-5

**Published:** 2005-06-29

**Authors:** Alessandro Grapputo, Tomi Kumpulainen, Johanna Mappes, Silja Parri

**Affiliations:** 1 Department of Biological and Environmental Science, University of Jyväskylä, P.O. Box 35, FI-40014 Jyväskylä, Finland

## Abstract

**Background:**

Despite the two-fold cost of sex, most of the higher animals reproduce sexually. The advantage of sex has been suggested to be its ability, through recombination, to generate greater genetic diversity than asexuality, thus enhancing adaptation in a changing environment. We studied the genetic diversity and the population structure of three closely related species of bag worm moths: two strictly sexual (*Dahlica charlottae *and *Siederia rupicolella*) and one strictly asexual (*D. fennicella*). These species compete for the same resources and share the same parasitoids.

**Results:**

Allelic richness was comparable between the sexual species but it was higher than in the asexual species. All species showed high heterozygote deficiency and a large variation was observed among *F*_IS _values across loci and populations. Large genetic differentiation was observed between populations confirming the poor dispersal ability of these species. The asexual species showed lower genotype diversity than the sexual species. Nevertheless, genotype diversity was high in all asexual populations.

**Conclusion:**

The three different species show a similar population structure characterised by high genetic differentiation among populations and low dispersal. Most of the populations showed high heterozygote deficiency likely due to the presence of null alleles at most of the loci and/or to the Wahlund effect. Although the parthenogenetic *D. fennicella *shows reduced genetic diversity compared to the sexual species, it still shows surprisingly high genotype diversity. While we can not totally rule out the presence of cryptic sex, would explain this high genotype diversity, we never observed sex in the parthenogenetic *D. fennicella*, nor was there any other evidence of this. Alternatively, a non-clonal parthenogenetic reproduction, such as automictic thelytoky, could explain the high genotypic diversity observed in *D. fennicella*.

## Background

Parthenogenetic females have a two-fold advantage over sexual females because they produce only the fecund sex while sexual females produce also males. The elimination of sex is predicted whenever the two strategies compete unless there are factors that overcome this disadvantage. Nevertheless, most of the higher animals reproduce sexually[1]. This leads to a fundamental question which continues to puzzle evolutionary biologists: how is sex maintained? A large body of theories seek to explain the maintenance of sex [2-7].

Advantages of sexual reproduction arise from genetic recombination in cross-fertilisation, which purges deleterious mutation and increases genetic variability in the population [8-10], enhancing adaptation in a changing environment. The idea that sexual reproduction and recombination may be favoured in changing environments has been the subject of several papers [11-15]. If a trait is subjected to stabilising selection, genetic variability introduces a genetic load as a consequence of the produced phenotypes that deviate from the optimum [16]. However, in a varying environment that exerts directional selection on a trait, genetic variability is essential because the response to selection will be proportional to the additive genetic variance in the population [15]. Under the mutation accumulation theory, the persistence of asexual lineages is more problematic unless asexuals are able to minimise the competition with sexuals through high dispersal rates [17].

Under the Red Queen hypothesis for sex [18], we should expect that heavy directional selection exerted by parasites can favour greater genetic variability in host populations. Parasites are more likely to infect the most common genotypes while rare genotypes, produced by sexual females, may escape infection [19-23]. Asexual reproduction is expected to be an unstable long term strategy since asexual females can only generate offspring with new genotypes through mutation.

The parasite hypothesis relies on several critical assumptions: the all-else-equal assumption and assumptions about the population structure and the genetic diversity of sexual and asexual populations [24]. The all-else-equal assumption (e.g. production of equal number and viability of offspring) depends on: how the asexuals originate, the type of parthenogenesis, and the degree of polyploidy of the asexuals [24,25]. The difference in population genetic structure between competing sexuals and asexuals may determine difference in the parasite infection load. Asexual hosts can persist in the long term, even in the presence of parasites, if they out-disperse their parasites [26,27]. The parasite hypothesis also assumes that sexual populations harbour higher levels of genetic diversity than asexual populations. The parasite hypothesis does not select for sex per se, but for diversity [28]. Thus, high clonal diversity could erode any advantage of sex. Howard and Lively [29] theoretically showed that host-parasite coevolution could lead to the accumulation of clones with different resistance genotypes and, in turn, to the elimination of sexual populations.

Few systems with coexisting sexual and asexual competitors are known. So, comparisons of genetic diversity between coexisting sexual and asexual populations are scarce: e.g. the freshwater snails, *Potamopyrgus antipodarum *[7,20] and genus *Campeloma *[17,24] and the aphid *Rhopalosiphum padi *[30]. Additional comparisons are needed to further evaluate the parasite hypothesis for sex.

Bag worm moths (Lepidoptera: Psychidae) provide an attractive case for investigating the coexistence of sexual and asexual reproduction in the same locations. In Lepidoptera, parthenogenetic reproduction is very rare. However, in the family Psychidae and especially among *Dahlica *species, parthenogenesis seems to have evolved several times [31,32]. A parthenogenetic (*Dahlica fennicella*, Suomalainen 1980) and two sexual species (*Siederia rupicolella*, Sauter 1954 and *D*. *charlottae*, Meier 1957) are common in Finland and often coexist in the same habitat. In these small insects (3–6 mm), adult females are always wingless, sessile and incapable of dispersing. Males are always winged but their dispersing ability is very limited and they can only fly short distances (between 10 and 100 m). Life cycle from egg to adult takes from one to two years, but the adults only live 3–6 days [33]. *S. rupicolella *and *D. fennicella *are very difficult to separate from each other and the only distinctive characters are their reproductive mode and genetic markers [32,34]. In central Finland, these bag worm moth species occur patchily in wooded habitats. The proportion of sexually and parthenogenetically reproducing species varies between locales from the total absence of the sexual species to only their presence.

Psychid larvae are often infected by at least two common species of Hymenopteran parasitoids, e.g. *Orthizema *spp. [31,34]. Kumpulainen *et al*. [34] found a strong positive correlation between parasite prevalence and the occurrence of sexual reproduction in Finnish bag worm moth populations for three consecutive years. *S. rupicolella *(sexual) was more abundant where parasitoids were more common, whereas *D. fennicella *(asexual) was more common in localities where parasitoids were scarce or absent. This result could argue in favour of the parasite hypothesis for the maintenance of sex. In light of this previous result, we investigated the genetic variability and the population structure of three closely related species of bag worm moths, two strictly sexual (*Siederia rupicolella *and *Dahlica charlottae*) and one parthenogenetic (*D. fennicella*) using isozyme variation.

## Results

### Genetic variability

Thirteen loci from ten isozymes were detected (Table 1). All were polymorphic in the two sexual species whereas twelve were polymorphic in the asexual species, with only fumaric acid (*FUM*) being monomorphic. No more than two bands were observed at all loci in the asexual *D. fennicella*, thus it is possible that this species is not tetraploid as are its relatives *D. lichenella *and *D. triquetrella *(tetraploid race) [35]. Ewens-Watterson [36] and Chakraborty's [37] test of neutrality indicated that the polymorphism observed, at the scale investigated, can confidently be assumed to be neutral.

**Table 1:** Isoenzymatic loci scored for *Siederia rupicolella*, *Dahlica charlottae* and *D. fennicella*. Recipes for buffers used are found at

Enzyme	E.C.*	Locus	Buffer
- Isocitrate Dehydrogenase	1.1.1.42	*IDH*	Phosphate 0.02 M pH 7.0
- Diaphorase	1.1.1.40	*DIA*	Phosphate 0.02 M pH 7.0
- Glucose-6-Phosphate Dehydrogenase	1.1.1.49	*G6PDH*	Tris-Maleate-EDTA- MgCl_2 _0.2 M pH 7.8
- 6-Phosphogluconate Dehydrogenase	1.1.1.44	*6PGDH*	Tris-Maleate-EDTA- MgCl_2 _0.2 M pH 7.8
- Aspartate Aminotransferase	2.6.1.1	*GOT*	Citrate Phosphate 0.04 M pH 6.4
- Phosphoglucomutase	5.4.2.2	*PGM*	Tris-Maleate-EDTA- MgCl_2 _0.2 M pH 7.8
- Malate Dehydrogenase NADP+	1.1.1.40	*ME1* *ME2* *ME3*	Tris Maleate 0.1 M pH 5.3
- Malate Dehydrogenase	1.1.1.37	*MDH MDH2*	Tris-Maleate-EDTA- MgCl_2 _0.2 M pH 7.8
- Glucose-6-Phosphate Isomerase	5.3.1.9	*GPI*	Tris-Maleate-EDTA- MgCl_2 _0.2 M pH 7.8
- Fumarate Hydratase	4.2.1.2	*FUM*	Tris Maleate 0.1 M pH 5.3

* Enzyme Commission Number

The estimates of genetic variability are shown in Table 2. Sufficient sampling of all three species in each population for population genetic analyses was not possible. There were two reasons for this; 1) although all three species were present to some extent in each location, they were not all abundant, particularly *D. fennicella*, and 2) females of the sexual species *S. rupicolella *are difficult to separate from *D. fennicella *without observing their mating behaviour. While sexual females secrete pheromones to attract males and do not lay eggs before mating, parthenogenetic females lay eggs in their larval case immediately after hatching from pupa. Species determination for sexual females was performed by experimental mating with a male. Because the adults are very short lived, females can mated with males hatching only few days apart restricting the sample size.

**Table 2:** Sample sizes, average number of alleles per locus, allelic richness, proportion of different genotypes (k), Simpson's index (D) and Evenness (E) (D and E calculated for asexual *D. fennicella *only), observed heterozygosity (H_o_), gene diversity (H_s_), and *F*_IS _are presented for each population of each sexual species. In the last column are presented the averaged values per species and the *F*_ST _values among populations.

	**Jyväskylä**															**Orimattila**					
*D. charlottae*	**Kö**		**Lv1**	**Lv2**	**Lv3**	**Lv4**		**Muu**	**Hj**		**Pih**	**Isa**	**Sip1**			**Sal**	**Tuk**	**Vilj**	**Vill**		

N	18		11	9	14	10		12	16		14	7	7			14	9	13	11		11.786
N. alleles ± (S.E.)	3.846 (1.039)		3.077 (0.485)	2.692 (0.537)	2.923 (0.611)	3.000 (0.553)		3.154 (0.761)	3.692 (0.888)		3.077 (0.564)	2.154 (0.281)	2.692 (0.511)			3.462 (0.719)	3.000 (0.707)	3.231 (0.649)	3.538 (0.860)		5.6154 (1.4485)
Allelic richness	3.029		2.871	2.543	2.593	2.765		2.790	3.049		2.702	2.119	2.582			2.877	2.804	2.884	3.114		2.766
k	1.000		1.000	0.889	0.929	1.000		1.000	1.000		0.786	1.000	1.000			1.000	1.000	1.000	1.000		0.972
Ho ± (S.E.)	0.297 (0.077)		0.307 (0.093)	0.249 (0.087)	0.295 (0.095)	0.308 (0.081)		0.245 (0.075)	0.266 (0.070)		0.251 (0.070)	0.374 (0.109)	0.393 (0.112)			0.248 (0.063)	0.274 (0.065)	0.284 (0.087)	0.285 (0.076)		0.2955 (0.1046)
Hs ± (S.E.)	0.469 (0.107)		0.548 (0.086)	0.451 (0.102)	0.437 (0.114)	0.508 (0.110)		0.447 (0.107)	0.505 (0.127)		0.502 (0.093)	0.405 (0.087)	0.425 (0.102)			0.524 (0.080)	0.468 (0.121)	0.528 (0.099)	0.558 (0.093)		0.4851 (0.0841)
*F*_ST _(P)								0.168 (<0.001)										0.154 (<0.001)			0.1530 (<0.001)

*S. rupicolella*		**Kv**		**Lv2**		**Lv4**	**Lv5**	**Muu**	**Hj**				**Sip1**						**Vill**	**Pen**	

N		10		14		10	9	14	17				26						11	11	13.556
N. alleles ± (S.E.)		2.846 (0.337)		3.000 (0.358)		3.000 (0.340)	2.385 (0.368)	3.231 (0.426)	3.385 (0.549)				3.692 (0.634)						2.538 (0.332)	2.308 (0.208)	2.932 (0.815)
Allelic richness		2.742		2.794		2.783	2.275	2.914	2.988				3.001						2.480	2.198	2.686
k		1.000		0.929		1.000	1.000	0.929	1.000				1.000						0.909	0.909	0.964
Ho ± (S.E.)		0.364 (0.084)		0.327 (0.086)		0.247 (0.063)	0.253 (0.071)	0.312 (0.084)	0.343 (0.092)				0.266 (0.068)						0.303 (0.096)	0.192 (0.073)	0.290 (0.072)
Hs ± (S.E.)		0.534 (0.057)		0.512 (0.072)		0.507 (0.057)	0.380 (0.073)	0.492 (0.062)	0.526 (0.069)				0.501 (0.066)						0.477 (0.058)	0.399 (0.066)	0.481 (0.049)
*F*_ST _(P)								0.114 (<0.001)										0.116 (<0.001)			0.101 (>0.001)

*D. fennicella*										**Hn**	**Pih**	**Isa**	**Sip1**	**Sip2**	**Sip3**						

N										9	13	17	18	21	8						14.333
N. alleles ± (S.E.)										1.923 (0.211)	2.692 (0.365)	2.615 (0.241)	2.385 (0.350)	2.769 (0.411)	1.692 (0.208)						3.615 (0.488)
Allelic richness										1.692	2.123	2.126	1.955	2.125	1.611						1.939
k										1	1	0.824	0.778	0.667	0.500						0.795
D										9	13	9.966	11.571	10.756	3.556						9.642
E										1	1	0.712	0.827	0.964	0.889						0.899
Ho ± (S.E.)										0.376 (0.117)	0.383 (0.115)	0.363 (0.122)	0.443 (0.122)	0.436 (0.133)	0.375 (0.130)						0.397 (0.116)
Hs ± (S.E.)										0.308 (0.069)	0.458 (0.067)	0.448 (0.058)	0.379 (0.0793)	0.466 (0.066)	0.279 (0.076)						0.390 (0.033)
*F*_ST _(P)								0.213 (<0.001)													0.213 (<0.001)

Allelic richness ranged from 1.61 in *D. fennicella *to 3.11 in *D. charlottae*. The expected heterozygosity (Hs) ranged from 0.279 in *D. fennicella *to 0.558 in *D. charlottae*. Allele richness and gene diversity (Hs) were similar in the two sexual species (1000 permutation: P = 0.488 and P = 1.00, respectively). Both sexual species harbour significantly higher allele richness and Hs than the asexual species (permutation tests: *D. charlottae *vs. *D. fennicella *P = 0.002 and P = 0.007, respectively and *S. rupicolella *vs. *D. fennicella *P = 0.012 and P = 0.012, respectively). As expected, the proportion of different genotypes (k) was close to 1 in the sexual populations and no differences were observed between the two species (Mann-Whitney test U_14,9 _= 50.5, P = 0.439). The proportion of different clones (k) was also high in the asexual species, ranging from 0.5 to 1 in the different populations; however it was significantly lower than in *D. charlottae *and almost significantly lower than in *S. rupicolella *(Mann-Whitney test U_14,6 _= 18, P = 0.020 and U_9,6 _= 13, P = 0.082). Evenness was very similar among asexual populations and it was very close to 1 because of the high genotype diversity. Significant deviation from the Hardy-Weinberg equilibrium was observed in most of the loci in all three species. In the sexual species, this deviation was due to heterozygote deficiency. The *F*_IS _values, over all loci indicated a significant deficiency of heterozygotes in all populations of both sexual species with the exception of Isosaari (Isa) and Sippulanniemi 1 (Sip 1) populations of *D. charlottae *(Table 3). In *D. fennicella*, instead, only two populations showed heterozygosity deficiency (Table 3). A large variation in *F*_IS _values was observed across all loci and populations in all three species with the exception of MDH2 for which no heterozygote individuals were ever observed (Table 3). The exclusion of this locus, however, did not change any of our results.

Null alleles might cause deviation from H-W proportion. The presence of null alleles at many of the loci analysed is strongly suggested by the significant correlation of the *F*_IS _values between the two sexual species (r_s _= 0.93, n = 12, P < 0.001) and between *D. charlottae *and *D. fennicella *(r_s _= 0.774, n= 11, P = 0.005). Calculation of the frequency of null alleles with the methods of Brookfield [38] indicate that null alleles are present in high frequencies in most of the loci in all three species (Table 3).

**Table 3:** *F*_IS _values and frequency of null alleles for each population of the three species at each locus. Null alleles frequencies (a*) were calculated with the method of Brookfield [38].

			*DIA*	*FUM*	*G6PDH*	*GOT*	*GPI*	*IDH*	*MDH*	*MDH2*	*ME1*	*ME2*	*ME3*	*6PGDH*	*PGM*	All
***D. charlottae***																

**Jyväskylä**	**Kö1**	*F*_IS_	-0.278	NA	0.141	0.721	0.619	0.115	0.256	1	0.368	1	1	0.211	0.011	0.367
		a*	0	0	0.027	0.257	0.215	0.038	0.092	0.363	0.116	0.172	0.095	0.042	0	
	**Lv1**	*F*_IS_	-0.653	NA	-0.297	0.773	0.828	-0.071	0.877	1	0.429	1	1	0.100	0.279	0.441
		a*	0	0	0	0.202	0.265	0	0.348	0.410	0.145	0.346	0.284	0.014	0.094	
	**Lv2**	*F*_IS_	-0.556	1	0.276	0.000	0.636	-0.297	0.429	1	1	1	NA	-0.143	0.689	0.448
		a*	0	0.165	0.069	0	0.130	0	0.133	0.331	0.308	0.417	0	0	0.258	
	**Lv3**	*F*_IS_	0.034	NA	-1	0.840	0.576	-0.243	0.425	1	NA	1	NA	0.407	0.278	0.326
		a*	0	0	0	0.243	0.214	0	0.146	0.252	0	0.417	0	0.092	0.097	
	**Lv4**	*F*_IS_	-0.292	NA	-0.125	0.385	0.717	-0.021	0.217	1	1	0.509	0	1	0.223	0.393
		a*	0	0	0	0.128	0.271	0	0.070	0.383	0.153	0.169	0	0.319	0.067	
	**Muu**	*F*_IS_	0.165	NA	-0.100	0.681	0.701	0.079	0.133	1	1	1	1	0.429	0.471	0.481
		a*	0.044	0	0	0.215	0.231	0.014	0.038	0.379	0.133	0.217	0.133	0.089	0.186	
	**Hj**	*F*_IS_	0.167	NA	-0.189	0.605	0.683	0.025	0.534	1	0.659	1	0	0.556	0.544	0.474
		a*	0.045	0	0	0.220	0.285	0	0.221	0.418	0.095	0.105	0	0.173	0.229	
	**Pih**	*F*_IS_	-0.241	NA	0.226	0.658	0.506	0.158	0.477	1	0.871	1	1	-0.294	0.577	0.501
		a*	0	0	0.049	0.105	0.139	0.052	0.195	0.384	0.294	0.290	0.333	0	0.223	
	**Isa**	*F*_IS_	-1	NA	0	0	0.091	-1	0.143	1	-0.200	1	0.750	-1	-0.714	-0.113
		a*	0	0	0	0	0.006	0	0.021	0.2899	0	0.1967	0.238	0	0	
	**Sip1**	*F*_IS_	0.615	NA	-0.091	-0.333	-0.161	-0.333	0.300	1	0.647	NA	1	-0.667	-0.448	0.076
		a*	0.152	0	0	0	0	0	0.059	0.3725	0.158	0	0.197	0	0	

**Orimattila**	**Sal**	*F*_IS_	0.393	-0.040	0.189	0.589	0.482	-0.026	0.816	1	0.662	0.769	1	0.154	0.564	0.527
		a*	0.121	0	0.047	0.223	0.179	0	0.338	0.351	0.191	0.178	0.332	0.030	0.133	
	**Tuk**	*F*_IS_	-0.333	NA	-0.200	0.632	0.575	0.127	0.744	1	-0.067	1	NA	-0.091	0.571	0.415
		a*	0	0	0	0.211	0.223	0.029	0.315	0.331	0	0.165	0	0	0.220	
	**Vilj**	*F*_IS_	0.208	NA	0.294	0.222	0.177	-0.200	0.866	1	1	1	1	0.353	0.392	0.463
		a*	0.066	0	0.061	0.073	0.048	0	0.311	0.362	0.327	0.321	0.124	0.103	0.155	
	**Vill**	*F*_IS_	-0.135	1	0.438	0.615	0.870	0.067	0.512	1	0	1	1	0.043	0.681	0.489
		a*	0	0.142	0.130	0.232	0.335	0.009	0.195	0.284	0	0.142	0.332	0	0.289	

***S. rupicolella***																

**Jyväskylä**	**Kv**	*F*_IS_	-0.800	NA	0.250	0.554	0.419	-0.268	0.534	1	0.442	0.852	0.542	0.351	-0.184	0.318
		a*	0	0	0.067	0.198	0.091	0	0.185	0.296	0.159	0.319	0.190	0.096	0	
	**Lv2**	*F*_IS_	-0.307	NA	0.559	0.458	0.780	-0.279	-0.078	1	0.579	0.882	NA	0.100	0.242	0.361
		a*	0	0	0.192	0.165	0.178	0	0	0.413	0.216	0.330	0	0.014	0.080	
	**Lv4**	*F*_IS_	-0.091	0	0.617	0.664	1	0.338	0.820	1	0.471	1	0	-0.207	-0.154	0.513
		a*	0	0	0.189	0.223	0.390	0.120	0.269	0.398	0.148	0.296	0	0	0	
	**Lv5**	*F*_IS_	0.067	NA	0.368	-0.091	0.573	-0.085	-0.067	1	0.818	NA	NA	0.478	0.190	0.333
		a*	0	0	0.087	0	0.172	0	0	0.257	0.283	0	0	0.137	0.045	
	**Muu**	*F*_IS_	0.007	NA	-0.132	0.480	0.623	-0.226	0.182	1	0.196	1	1	-0.176	0.507	0.367
		a*	0	0	0	0.178	0.158	0	0.063	0.364	0.033	0.333	0.290	0	0.170	
	**Hj**	*F*_IS_	-0.333	1	0.364	0.202	0.615	-0.200	0.813	1	0.509	0.652	NA	0.336	-0.049	0.347
		a*	0	0.172	0.111	0.072	0.251	0	0.299	0.386	0.177	0.088	0	0.096	0	
	**Sip1**	*F*_IS_	-0.090	NA	-0.154	0.301	0.788	0.001	0.708	1	0.091	1	0.928	-0.091	0.358	0.468
		a*	0	0	0	0.085	0.321	0	0.303	0.393	0.018	0.316	0.316	0	0.132	

**Orimattila**	**Vill**	*F*_IS_	-0.800	NA	-0.047	1	1	-0.125	0.612	1	-0.296	1	1	-0.2000	0.474	0.366
		a*	0	0	0	0.339	0.316	0	0.240	0.316	0	0.153	0.316	0	0.142	
	**Pen**	*F*_IS_	0.438	NA	0.217	0.091	1	-0.524	0.680	1	0	1	1	NA	-0.026	0.518
		a*	0.130	0	0.054	0.014	0.332	0	0.225	0.373	0	0.301	0.332	0	0	

***D. fennicella***																

**Jyväskylä**	**Hn**	*F*_IS_	0.304	NA	-1	0	-0.600	-0.091	0.448	1	NA	NA	NA	-1	-1	-0.222
		a*	0.093	0	0	0	0	0	0.108	0.331	0	0	0	NA	0	
	**Pih**	*F*_IS_	0.268	NA	-1	1	0.200	0.050	0.553	1	0.351	1	1	-1	-0.581	0.145
		a*	0.110	0	0	0.321	0.000	0.058	0.301	0.381	0.256	0.299	0	0	0	
	**Isa**	*F*_IS_	0.657	NA	-1	1	-0.333	0.185	0.780	1	0.840	1	NA	-0.800	-1	0.208
		a*	0.058	0	0	0.333	0.067	0.008	0.205	0.279	0.067	0.233	0.172	0	0	
	**Sip1**	*F*_IS_	-0.732	NA	-1	1	-0.308	-0.244	0.532	1	NA	-0.097	NA	-0.846	-1	-0.168
		a*	0	0	0	0.095	0	0	0.207	0.436	0	0	0	0	0	
	**Sip2**	*F*_IS_	0.650	NA	-1	0.840	-0.772	-0.159	0.162	1	1	1	NA	-1	-1	0.057
		a*	0.218	0	0	0.185	0	0	0.052	0.393	0.329	0.329	0	0	0	
	**Sip3**	*F*_IS_	0.391	NA	-1	NA	NA	-1	-0.029	1	NA	NA	NA	-1	-1	-0.345
		a*	0.091	0	0	0	0	0	0	0.347	0	0	0	0	0	

### Population differentiation

**Figure 2:**
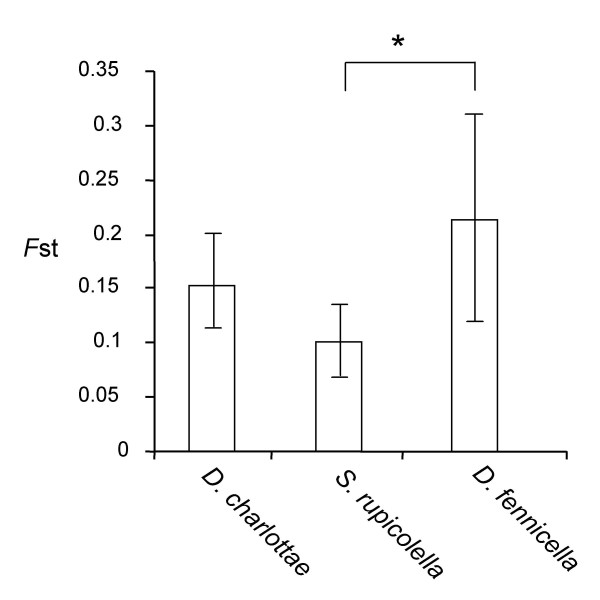
Mean *F*_ST _of each species and its 95% confidence interval obtained with bootstrap over loci. Asterisk indicates 5% significant level.

The overall differentiation among populations, *F*_ST_, was high and significantly different from zero in all three species (Table 2), which is an indication of strong population structure. Mean *F*_ST _values across loci and the 95% confidence interval (bootstrap over loci) are shown in Figure 2. The overall differentiation was significantly different in the three species. While the mean *F*_ST _value of *D. fennicella *was not different from that of *D. charlottae*, it was significantly higher than that of *S. rupicolella*. Hierarchical analyses of molecular variance (AMOVA) indicated that the two areas, Jyväskylä and Orimattila, did not differ from each other for both sexual species. In *D. charlottae *the percentage of variance among sites was 1.61, P = 0.12, while it was 16.07, P < 0.001 among populations within sites. In *S. rupicolella *the percentage of variance among sites was 0.85, P = 0.27, while it was 11.45, P < 0.001 among populations within sites. We did not observe isolation by distance (Mantel test between *F*_ST_/(1-*F*_ST_) and the natural logarithm of the geographical distance) in any species at either of the sites: *D. charlottae *(Jyväskylä R = -0.257, P = 0.089; Orimattila R = 0.353, P = 0.512), *S. rupicolella *(R = 0.222, P = 0.338) and *D. fennicella *(R = - 0.300, P = 0.277).

Sixty five different genotypes were detected among the 86 samples of *D. fennicella*. Only two of them were shared among different populations. One genotype was shared between Sippulanniemi 1 (Sip1) and Huviniemi (Hn) while the other was shared between Sippulanniemi 3 (Sip3) and Pihta (Pih). In both cases the two populations are geographically distant, and three populations in the same area (Sip1, 2 and 3 and Hn, Pih and Isa, respectively) did not share any genotypes.

## Discussion

Analysis of isozyme variation in three species of psychid moths revealed that the genetic diversity of the sexual species *D. charlottae *and *S. rupicolella *is higher than that of the asexual species *D. fennicella*. Allele richness, gene and genotypic diversity were also higher in the sexual species than in the asexual species. Higher genotype diversity in sexual than in asexual populations is most likely the logical result of recombination and was an expected result. The sexual populations also showed higher allele diversity, which is a more intriguing outcome [30]. One possible explanation for this difference is that the asexual lineage retained only a portion of the diversity of its sexual ancestor. Alternatively, a lower per locus diversity in the asexual species could reflect lower population sizes compared to the sexual species. Asexual *D. fennicella *is, in general, rarer than the sexual species, although it was the most abundant species in some locations. A lower population size is also suggested by the higher differentiation (*F*_ST_) among *D. fennicella *populations than among sexual populations.

Surprisingly, parthenogenetic *D. fennicella *showed a considerable amount of genotype diversity, with 65 different genotypes detected among 86 individuals. This was in contrast with a previous analysis with allozyme markers which found limited diversity among samples of *D. fennicella *[39]. This amount of genotype diversity was higher than that recently observed in *Potamopyrgus *snails [20] and that reported in animals (reviewed in[40]) and in plants (reviewed in [41]) in the previous allozymes literature. Interestingly, clonal lineages of *D. fennicella *were mostly restricted to single populations. Only two genotypes were shared among distant populations. The lack of a common broadly adapted haplotype spread over different populations is in conflict with the hypothesis of the general-purpose-genotype [42]. Instead, adaptation to different microclimates or other specific environmental conditions of these locales could explain the presence of many different genotypes, as suggested by Vrijenhoek's [43] frozen niche variation hypothesis. However, we found no significant differences in morphology, size and life-history characters between two different *D. fennicella *populations that would reflect ecological specialisation [34]. Although several studies have reported allozymes as not neutral (reviewed in [44,45]), in our study there were no indications that they deviate from neutrality, thus these markers are expected to be subjected more to drift than to selection. High genotypic diversity could indicate the presence of cryptic sex in the parthenogenetic species. Although we cannot completely rule out this hypothesis, we never observed sex in the species. All parthenogenetic females lay eggs immediately after hatching from pupa and never show the characteristic behaviour of sexual females when they secrete pheromones to attract potential mates (Kumpulainen et al. 2004). Moreover, mitochondrial sequences from sexual and asexual females clearly indicate these are two different species (Grapputo et al. 2005). This high genotypic diversity could also be explained by alternative types of parthenogenesis involving recombination, such as the automictic thelytoky [46].

High clonal diversity and the observed distribution of different clones could be the result of a restricted dispersal capacity and the fragmentation of suitable habitats for these psychid moths. Large differentiation was also observed among populations of diploid parthenogenetic *D. triquetrella *in the Alps but not among tetraploid populations of the same species in Finland [47]. The same pattern, however, could be explained by an extinction-colonisation process associated with a long persistence of the populations, which would explain the high intrapopulation diversity. Large genetic differentiation among populations was also observed in both the sexual species, *D. charlottae *and *S. rupicolella*, which is consistent with their extremely low ability for active dispersal (see also [31]) and the patchy distribution of suitable habitats. Nevertheless, psychid moths sometimes colonise new areas as suggested by the absence of *D. charlottae *in the Isosaari population in 1999 and its presence in 2000 (T. Kumpulainen, personal observation). Most probably, dispersal between different populations is a relatively rare event taking place as passive aerial dispersal of very small larvae [31]. The large genetic differentiation among *D. charlottae *and *S. rupicolella *populations is in contrast with the data obtained for populations of sexual *D. triquetrella *in the Alps by Lokki *et al*[47], where allelic frequencies were described as homogeneous among populations, although rigorous tests of population differentiation were not carried out.

The observed proportion of heterozygotes was not different between the two sexual species *D. charlottae *and *S. rupicolella *(0.29) and was very similar to that previously observed in another sexual species *D*. *triquetrella *(0.23) [47]. The level of heterozygosity was also highly similar among populations in both sexual species. *D. charlottae *and *S. rupicolella*, in contrast to *D. triquetrella*, were not in HW equilibrium for most of the loci and populations. Heterozygote deficiency has been widely reported in allozyme surveys of natural populations of marine invertebrates (reviewed in [48,49]) and also in fishes (e.g. [50,51]), amphibians and reptiles (reviewed in [52]). Alternative hypotheses have been advanced to explain such heterozygote deficiencies [48,49,53]. The high heterozygosity deficiency in all three species of bag worm moths could be explained by null alleles. The high variation across loci in *F*_IS _values correlate among species and the methods of Brookfield [38] for the calculation of null alleles frequencies strongly suggest that most of the loci in the three species are affected by null alleles. Most populations of sexual psychid moths are small, consisting of just 30 to 100 individuals. Suitable forest patches are also small and isolated. Moreover, females are apterous and unable to disperse. When sexual females emerge from pupae they quickly start to secrete pheromones to attract males. Once emerged, males respond promptly to the female pheromones because they have a very short adult life span (about 10 hours). Therefore, copulation most likely occurs between emerging adults that are both spatially and temporally close. This could create substructured populations and a Wahlund effect, both spatial and temporal, which could maintain a high number of alleles in the population but increase the homozygosity [54].

## Conclusion

In summary, the three different moth species show a similar population structure characterised by high genetic differentiation among populations and low dispersal. The parthenogenetic *D. fennicella *shows reduced genetic diversity compared to the sexual species but still shows high genotype diversity that could indicate the presence of cryptic sex. All species show a very high heterozygote deficiency due to the presence of null alleles at most of the loci or to the Wahlund effect. DNA markers certainly need to be investigated to determine the causes of such heterozygote deficiency shown by the allozymes.

## Methods

### Source populations

**Figure 1:**
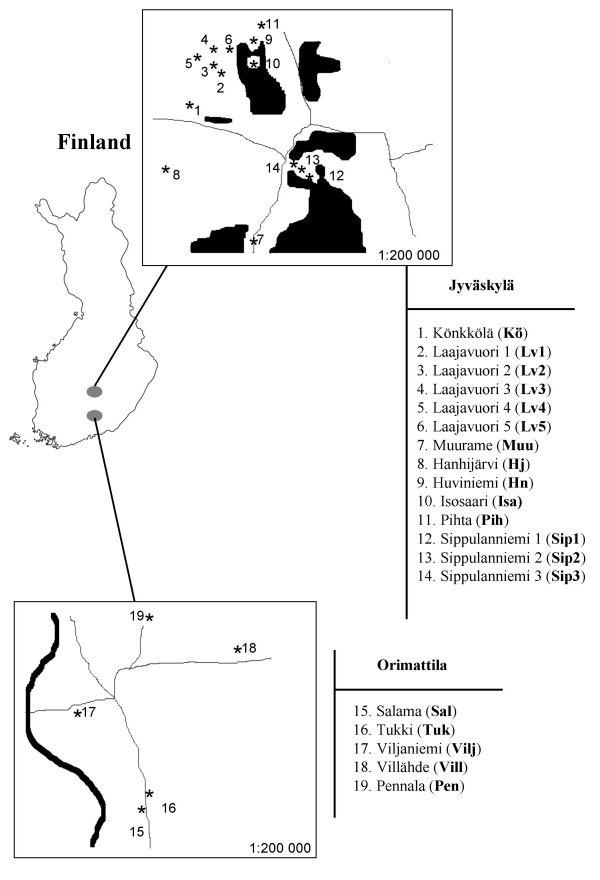
Map of the sampling sites in central Finland.

Two sexual species, *Siederia rupicolella *and *Dahlica charlottae*, and an asexual species, *D. fennicella *were sampled to study their genetic variability and population structure. Samples were collected in April 2000 from 20 different study areas of suitable forest type [34]. All areas were situated in central Finland, 15 of them around the city of Jyväskylä (62 °15 N', 25°43 E') and five close to the town of Orimattila (60 °49 N', 25 °40 E') (Figure 1). Study areas consisted of old forest patches, separated by meadows and fields and sometimes by human settlements. All study areas were dominated by mixed forests of Norwegian spruce (*Picea abies*) and silver birch (*Betula pendula*), many of them also contained Scotch pine (*Pinus sylvestris*). Final instar larvae of all three moth species climb on tree trunks to pupate and they can easily be caught by setting tape traps on tree trunks. Larvae remain stuck on the tape and they can later be collected. Each collected larva was taken to the laboratory and kept individually until hatching to adult, allowing us to determine the reproduction mode and identify the species [34]. Samples were subsequently frozen at -80°C until analysis.

### Electrophoresis

Frozen samples were squashed in 20 μl of grinding buffer (Tris-HCl 0.1 M pH = 8.0) and then applied to Titan^® ^III cellulose acetate plates (76 mm × 76 mm) using the Super Z-12 applicator Kit (Helena laboratories) following the method of Hebert and Beaton [55]. Electrophoresis was carried out at room temperature at 200 volts for 20–25 minutes in the appropriate buffer for each enzyme as indicated in Table 1. Of twenty-three enzymes tested, ten were polymorphic (listed in Table 1) from which a total of thirteen loci could be scored. Enzymes excluded from the analysis because they were monomorphic or unreadable were: *ACON *(EC 4.2.1.3), *AK *(EC 2.7.4.3), *ADH *(EC 1.1.1.1), *ALP *(EC 3.1.3.1), *ATT *(EC 2.6.1.1), *EST *(EC 3.1.1.1), HEX (EC 2.7.1.1), *LDH *(EC 1.1.1.27), *LAP *(EC 3.4.11.1), *MPI *(EC 5.3.1.8), *SOD *(EC 1.15.1.1), *α, α-Trehalase *(EC 3.2.1.28) and *SKDH *(EC 1.1.1.25).

### Data analysis

Tests of neutrality for each locus, population and species were carried out using the Ewens-Watterson test [36] with the software package Popgene [56]. The genetic diversity and population structure of each species were analysed using Fstat [57]. We tested the Hardy-Weinberg equilibrium (HW) for each locus and population by randomisation of alleles among individuals within populations. Significance levels were adjusted using the sequential Bonferroni correction for multiple comparisons [58]. For each population we estimated the number of alleles per locus, allelic richness [59], gene diversity (Hs) [60], observed heterozygosity (Ho) and *F*_IS _value. Frequency of null alleles per locus and population was estimated with the method of Brookfield [38] as implemented in Micro-Checker v.2.2.3 [61], which does not require detecting null allele homozygotes. Genotypic diversity (or clonal diversity in asexuals) within populations was determined simply as the proportion of different genotypes in the population *k *= *G*/*N*, where G is number of genotypes and N is the number of individuals in the population. For the asexual species, we also measured clonal diversity using Simpson's diversity index D = *1/∑p*_i_, where *pi *is the frequency of the i-th clone (Simpson, 1949). D varies from 1 (monoclonal population) to N if each individual carries a different genotype. This measure takes into account the frequency of clones, but it depends on the sample size, so we also calculated the evenness (E) of Simpson's index E = D/D_max_, which is constrained between 0 and 1. Population structure was assessed by calculating *F*_ST _[62] between populations and tested by permuting genotypes among samples because most of the populations were not in HW (as suggested in Fstat). Hierarchical analysis of molecular variance (AMOVA, [63]) including all populations and populations within the two areas (Jyväskylä and Orimattila) was performed with Arlequin ver. 2.000 [64]. If the differentiation between populations is due to isolation by distance, a positive correlation between genetic distance and geographical distance is expected. Isolation by distance was tested as suggested by Rousset [65] and a Mantel test was performed between populations in each site using Fstat.

## List of abbreviations

*ACON *= Aconitate Hydratase, *AK *= Adenylate Kinase, *ADH *= Alcohol Dehydrogenase, *ALP *= Alkaline Phosphatase, *AAT *= Amino Aspartate Transferase, *EST *= Carboxylesterase, HEX = Hexokinase, *LDH *= Lactate Dehydrogenase, *LAP *= Leucine Aminopeptidase, *MPI *= Mannose-6-Phosphate Isomerase, *SOD *= Superoxide Dismutase and *SKDH *= shikimate dehydrogenase.

## Authors' contributions

TK collected samples and performed most of the laboratory procedure with SP. AG and JM performed the analysis of the data and wrote the manuscript. All the authors contributed to the study.
